# A scoping review on the relationship between mental wellbeing and medical professionalism

**DOI:** 10.1080/10872981.2023.2165892

**Published:** 2023-01-09

**Authors:** Kamran Sattar, Muhamad Saiful Bahri Yusoff, Wan Nor Arifin, Mohd Azhar Mohd Yasin, Mohd Zarawi Mat Nor

**Affiliations:** a Department of Medical Education, School of Medical Sciences, Universiti Sains Malaysia, Kubang Kerian, Kelantan, Malaysia; b Biostatistics and Research Methodology Unit, School of Medical Sciences, Universiti Sains Malaysia, Kubang Kerian, Kelantan, Malaysia; c Department of Psychiatry, School of Medical Sciences, Universiti Sains Malaysia, Health Campus, Kubang Kerian, Kelantan, Malaysia

**Keywords:** Medical professionalism attributes, mental wellbeing, burnout, empathy, stress, relationship, scoping review

## Abstract

**Background:**

Mental wellbeing issues among medical students are common, and their relationship to medical professionalism is debated. Few studies have attempted to link such issues with undergraduate medical education. This review aimed to advance the knowledge on this matter by exploring the relationship between mental wellbeing and medical professionalism in undergraduate medical education.

**Methods:**

We collected the literature about mental wellbeing and medical professionalism (published from 1 January 1986 to 31 March 2021) from the Web of Science, PubMed, Scopus and ScienceDirect databases using the search terms ‘mental wellbeing’ and ‘medical professionalism’.We included all peer-reviewed articles in which mental wellbeing and medical professionalism in the undergraduate medical education context were the central topics regardless of the age range, nationality, race and gender of the participants.

**Results:**

From the 13,076 Iinitially found articles, 16 were included. These 16 articles were from nine countries in four different continents, which all together helped us find answer to our research question using extracted points relating to the main study themes (mental wellbeing and medical professionalism). Under theme 1 (mental wellbeing), six subthemes emerged: burnout, stress, depression, disappointment, depersonalisation and conscientiousness. Theme 2 (medical professionalism), on the other hand, had five subthemes: empathy, academic performance, compassion, unprofessional behaviour and professionalism. A significant inverse association was found between empathy and burnout. Academic performance was also related to burnout. At the same time, empathy was found to have a varied association with stress. Moreover, compassion was found to alleviate burnout and nurture professional gratification.

**Conclusion:**

The medical professionalism attributes were found to deteriorate as the mental wellbeing issues grow. This can harm medical students’ overall health, current learning abilities and future attitudes towards their patients. Explicit primary research is thus required to examine and intervene in the cause-effect relationship between medical professionalism and mental wellbeing.

## Introduction

With the current emerging awareness of the public about their rights as patients, the medical profession continuously seeks to improve the teaching of medical professionalism and professional behaviour among medical professionals and the evaluation of such in their daily practice.

Numerous definitions of professionalism have been reported. Professionalism was formerly viewed as an attitude [1]. Recently, however, several educationists have contested this view, defining professionalism instead as a collection of actions. It is one thing to know what constitutes professional behaviour; it is another to demonstrate professional behaviour consistently despite the pressures imposed on oneself by a busy practice with conflicting demands and multiple objectives [2]. As such, professionalism is an acquired skill that may be honed through time [3]. What is critical about this concept is that it is a range of actions rather than an all-or-nothing set of characteristics. Differentiating between unprofessional and professional physicians constitutes a departure from this paradigm. It is the possession of certain habits that makes one professional or not, and even the most skilled physician may exhibit behavioural lapses.

Medical professionalism is defined as ‘altruism, accountability, devotion to excellence, duty and devotion to service, honour, and respect for others’ [4]. It is also considered the foundation of the relationship between doctors and society [5,6]. Over the last quarter-century, there has been a widespread consensus that medicine and the society it serves have a social contract founded on the profession’s notion. Medical professionalism is a fundamental proficiency for medical students, and committed doctors as lifelong learners [7]. It must thus be included in the undergraduate curriculum [8]. Many educational programs have been advocated to promote the professional development of medical students, and such efforts have focused on keeping medical students from having unprofessional practices in the future. Despite these efforts, however, the unprofessional behaviour among medical students currently ranges from modest to severe [9].

Several definitions of empathy have been proposed in the literature to help achieve this balance in medical practice [10,11]. It is widely regarded as the cornerstone of humanistic medicine, which offers numerous advantages to both patients and doctors [12], and is also portrayed in the subject of medical professionalism as the right blend between emotional over-involvement and detachment. The quality of a doctor-patient relationship improves when the doctor tries to learn about his or her patients’ thoughts and feelings, shows interest, and shows concern. Diabetes patients with an empathetic physician had better control of their blood glucose and cholesterol levels than those whose physician lacked empathy [13]. During medical school, empathy levels decrease [14]. This has caused concerns among medical educators, as emphasised in the health education literature [15].

According to the World Health Organization, ‘health is a complete state of physical, mental, and social wellbeing and not merely the absence of disease or infirmity’ [16]. Although the definition of mental health varies by culture and is influenced by particular values, it is frequently regarded as equivalent to having an optimistic state of mind (e.g., cheerfulness, constructive emotional state) and engaging in constructive work in one’s life (e.g., intellectual persistence, satisfying affairs, sense of fitting in and contributing to society) rather than merely not being in low spirits (e.g., the absence of burnout does not mean that a person is emotionally well or flourishing) [16,17].

Mental health issues are becoming more prevalent among healthcare workers worldwide. Consequently, there has been an increasing focus on the mental suffering of medical students [18,19]. In a large study (using series of psychological self-report surveys, 12-items General Health Questionnaire (GHQ), the Maslach Burnout Inventory, and Course Stress Questionnaire) conducted in the United Kingdom, 30.6% of the first-year medical students who participated in the study, 30.6% of the fourth-year medical students and 21.9% of the fifth-year medical students scored above the borderline, suggesting that they were experiencing psychological distress [20]. Another study in Turkey, using (GHQ), the Spielberger State-Trait Anxiety Inventory, and the Beck Depression Inventory, found that 47.9% of the second-year medical students who participated in the study had emotional disorders, far higher than the percentages of economics students (29.2%) and physical education students (29.2%) [21]. According to a Malaysian study (using GHQ), 41.9% of the medical students in Malaysia who participated in the study were suffering from mental disorders [22].

One of the primary goals of medical schools is to prepare students to meet current and future national population healthcare needs. However, medical students typically require more time to complete their education than students from other disciplines [23], and the study of medicine is thought to be related with much higher levels of mental and physical stress than other academic disciplines [24]. Medical teaching can cause psychological issues in medical students. Such students’ ever-changing knowledge base and extensive and intensive training may have a long-term impact on their mental health and wellbeing. That is, such circumstances increase the chances that medical students will develop common mental diseases (e.g., depression, anxiety or stress) [25]. This can limit their ability to learn, and as they are expected to play a vital role in delivering health care in the future, it can affect the community they will serve by limiting their ability to practice professionalism.

### Association between medical professionalism and mental wellbeing

Medicine is fundamentally a human-service profession. In all human dealings, the practice of humanistic principles in general and of empathy in particular is crucial. As a result, the theme of professional development has been predominant in many academic medical publications and conference agendas in the last decade. According to the American Board of Internal Medicine [26], humanistic principles and empathy should also be promoted and evaluated as a fundamental instructive activity within the medical curriculum.

There has been much talk about mental wellbeing among medical students, and there have been several reports of decreases in students’ professionalism attributes, such as empathy [27,28], compassion and humanism [29], throughout medical school, which are thought to be a result of students’ reactions to the stresses they encounter in the classroom [18,28]. Interestingly, the degradation of empathy comes at a time when the focus of the curriculum is turning to patient care, where empathy is most needed [28]. As medical students progress through their medical education, up to three-quarters of them become progressively pessimistic about academic life and the medical profession [30]. The reasons for the changes in their attitude scores are unknown, but they could be related to the high attitude ratings at medical-school entry, an eventual loss of idealism on the part of the students as they go through medical studies and/or the unanticipated impact of the medical curriculum.

Although compromised mental wellbeing (e.g., burnout) is linked with a decline of professionalism [31], it is not certain if constructive mental health can boost medical professionalism (e.g., empathy). Compromised mental wellbeing can create a significant disconnect between the noble ideals to which professionals are expected to be committed and the reality of their day-to-day experience. Therefore, it is necessary to identify the mental wellbeing characteristics that have a relationship with medical professionalism, which may have the potential to affect or be affected by medical professionalism.

## Methods

This scoping review [32] was performed to summarise the contents of the current literature on the relationship between mental wellbeing and medical professionalism. We used the scoping review strategy to examine and collate a broader range of relevant literature as such method provides theoretical precision regarding a specific area of literature [33]. This aids in identifying the research gaps on the subject at hand [34,35]. Furthermore, such method is beneficial primarily because it can provide a deeper knowledge of the numerous complicated relevant issues that have not been researched systematically. We utilised the scoping review framework recommended by Arksey and O’Malley [34], which has five essential stages: (1) identifying the research question; (2) identifying relevant studies; (3) study selection; (4) charting the data; (5) collating, summarising, and reporting the results.

### Stage 1: Identifying the research question

This scoping review aimed to determine the relationship between mental wellbeing and medical professionalism in the context of undergraduate medical education by identifying and collating the available articles on such topic. Accordingly, the authors formulated the following research question to guide this scoping review, as its focus: What are the effects of undergraduate medical students’ mental wellbeing on their medical professionalism?

### Stage 2: Identifying relevant studies

**Figure 1.**
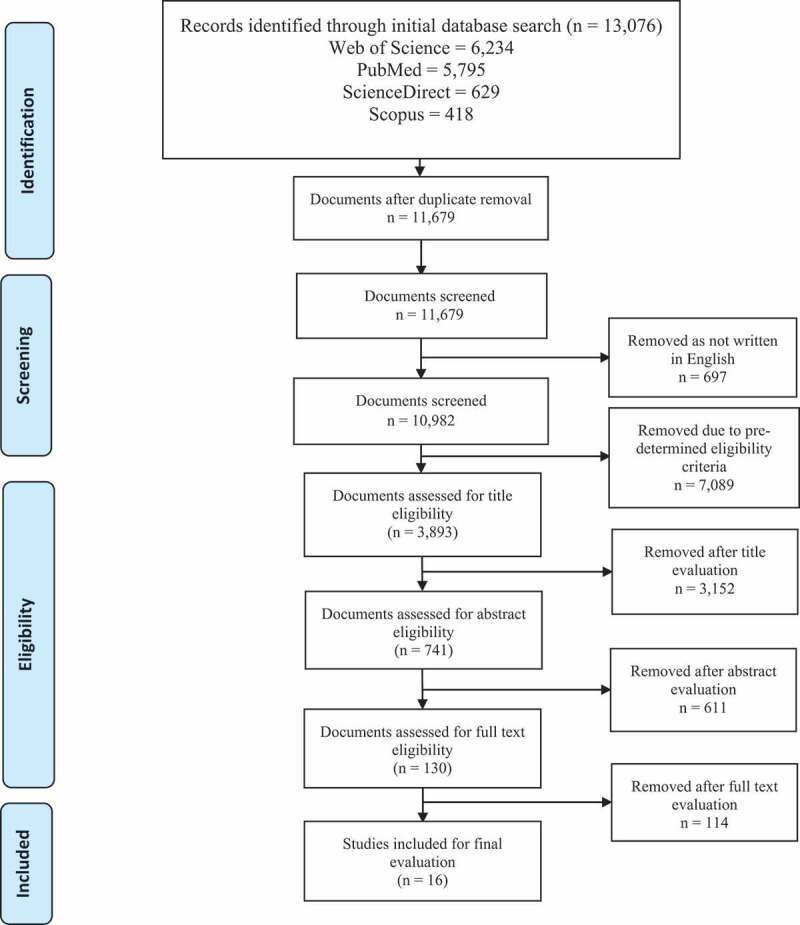
The scoping review consort diagram describes articles’ assortment for this review.

An electronic search was done for relevant articles published within the period from 1 January 1986 to 31 March 2021, using the Web of Science, PubMed, Scopus and ScienceDirect databases. We employed a three-step search for this review. To begin, in August 2021, we conducted a principal article search through Web of Science, PubMed, Scopus and ScienceDirect using all the established keywords and index terms. Secondly, we evaluated the titles and abstracts of the articles that we found. Thirdly, we combed through the reference lists of all the articles to find additional studies. We used well-defined heading terms to avoid missing any pertinent article, and we established an electronic search policy with the help of a librarian. We also conducted several test searches to optimise the search terms. Papers were searched using MeSH terms (although the MeSH terms varied slightly between the databases). For the list of the complete search terms that we used, see Appendix A. The following were the eventual search terms that we came up with using the Boolean operator ‘AND’: ‘mental wellbeing’ AND ‘medical professionalism’ AND ’‘medical education“AND ‘undergraduate’ AND’‘medical student.” Besides these, reference lists were searched for pertinent articles that might have been overlooked throughout the primary search. Google and Google Scholar were used to search for unpublished studies and grey literature. Following this secondary search, succeeding articles that met our eligibility criteria were considered for further investigation. The article selection method that we used was recorded using the Preferred Reporting Items for Systematic Reviews and Meta-Analysis (PRISMA) flowchart [36]. The flowchart documenting the data collection method can be viewed in Figure 1.

The Preferred Reporting Items for Systematic Reviews and Meta-Analysis (PRISMA) flow diagram for the selection of studies in this review - **PRISMA** [36]

### Stage 3: Study selection

The articles that directly matched or were similar to the keywords that we used were identified at this stage. A three-stage screening process was used (titles, abstracts and full text). Additionally, the contents of the entire articles were evaluated to determine the articles’ suitability for inclusion in the review. We considered articles suitable for inclusion in the review on the basis of the predetermined criteria for eligibility (see Table 1). Initially, 13076 papers were retrieved on the basis of the search terms. After removing the duplicates, we were left with 11,679 articles. We later selected 3,893 titles from among these on the basis of the set criteria, and we retrieved the study abstracts. After reviewing the suitability criteria, we then carefully assessed and chose 741 abstracts. On the basis of such abstracts, we selected 130 articles for full-text evaluation (Table 1). Two researchers collected the full-text articles [35]. A third researcher resolved the disagreements among the researchers on the choice of articles to include in the review. The inter-rater reliability of the two reviewers was high, with a 0.852 intraclass correlation coefficient, a 95% (0.823–0.8759) confidence interval and *p* < 0.001. Finally, we selected 16 articles for inclusion in the review and for data extraction and charting (see Figure 1). The official outcome of the exploration was transferred to the bibliographic software program EndNote (Clarivate Analytics, Philadelphia, PA).

**Table 1.** Study eligibility criteria.

Study Characteristics	Inclusion criteria	Exclusion criteria
Period	Published within the period from January 1, 1986, to March 31, 2021.	Outside these dates
(2) Language	English	Other than the English
(3) Title	With the overwhelming theme relating to mental wellbeing and medical professionalism	Not covering both or one of the two themes (mental wellbeing or medical professionalism)
(4) Abstract	Pertaining to the original research and available in a peer-reviewed journal Pertaining to studies conducted internationally or nationally Pertaining to studies within the context of undergraduate medical education Pertaining to studies with medical students and faculty as the participants Pertaining to studies that explored and highlighted mental wellbeing and medical professionalism	Non-peer-reviewed or non-original research Ideas, editorials, opinions, case reports and reviews Pertaining to postgraduate medical education Students and faculty from other health professions
(5) Full-text	With full-text articles available Elaborated the relationship between mental wellbeing and medical professionalism With a robust analysis of the results With a well-designed exploration intervention Reported the effects of mental wellbeing on medical professionalism and/or vice versa	Failed to elaborate the relationship between mental wellbeing and medical professionalism Not having a vigorous analysis of the results. Not reporting effects of mental wellbeing on medical professionalism and/or vice versa

The title, abstract and full text can be considered suitable if they coincide with the applicable study inclusion criteria.

### Stage 4: Charting the data

The a priori establishment of an organised charting method aided in extracting pertinent data from the studies included in the review [34]. Twelve randomly nominated articles were independently examined by two investigators using the data extraction form that they had formulated to help ensure that their data extraction approach would remain unchanged and relevant to the research question. The final document included two broad categories: article characteristics (e.g., publication year) and research focus area. The coding procedure was split into three stages: open coding, category creation and abstraction [37]. Three researchers firstly independently read each article and wrote notes about it and headings to identify its primary research area. Following that, the headings and notes were documented to come up with a set of preliminary codes. The collection was then improved cyclically, with similar codes blended into subcategories. Thereafter, we held debriefing meetings to finalise our explanations, make coding decisions and develop categories. Eventually, we conducted coding using NVivo [38], a qualitative data analysis software. Table 2 contains excerpts from the articles included in the review.

**Table 2.** List of original medical professionalism articles included as the selected studies for final evaluation with the retrieved information.

S. no.	Author	Year	Location	Aims/objective/background/ purpose (about MHDs)	Sample/study design/tools	Key findings related to the review questions
1	T Kötter, L Kiehn, KU Obst and E Voltmer [39]	2021	Germany	‘In this longitudinal study, we investigated the development of empathy during medical education and assessed [the] potential predictors of empathy at different time points in medical studies.’	43 medical students Longitudinal descriptive study Jefferson Scale of Empathy Student Version (JSE-S; available in the German language); Hospital Anxiety and Depression Scale in the German language (HADS-D); German language version of the Perceived Medical School Stress Scale (PMSS-D); AVEM (Arbeitsbezogenes Verhaltens-und Erlebensmuster [Work-Related Behaviour and Experience Pattern])	A negative association between the depression scores and the empathy scores was found. The study results confirm the earlier finding (negative relationship) regarding the association between depression and empathy at several time points in the study.
2	K Vidhukumar and M Hamza [40]	2020	India	“This study was planned to find the prevalence and correlates of burnout among [the] medical students at a Government Medical College in Kerala.”	375 medical students Cross-sectional survey-based study Copenhagen Burnout Inventory (CBI)	The learning environment and curriculum-related variables were not shown to be associated with burnout, as measured by the perceptions of teaching quality, academic performance and failure in the professional test.
3	Q Wang, L Wang, M Shi, X Li, R Liu, J Liu, M Zhu and H Wu [41]	2019	China	‘The objective of the present study is to explore the correlations of empathy and burnout with life satisfaction and the associated socio-demographic factors among Chinese undergraduate medical students.’	1,271 medical students Cross-sectional study Interpersonal Reactivity Index Chinese version (IRIC), Maslach Burnout Inventory modified Chinese version (MBI-MC), Satisfaction with Life Scale (SWLS) and socio-demographic characteristics	The medical students’ empathy levels decreased in four years, but their burnout levels remained nearly constant. Empathy was linked with pupils’ age and grade level while burnout was linked with maternal education.
4	JW Wahjudi, A Findyartini and F Kaligis [42]	2019	Indonesia	‘Empathy is critical for medical doctors, as it enables them to conduct good patient-centred care. Medical students are expected to learn this ability as part of their education and training.’	464 medical students Cross-sectional design 10-item version of the Perceived Stress Scale (PSS-10) translated into the Indonesian language; Jefferson Scale of Physician Empathy (JSPE) translated into Indonesian	The medical students’ stress levels peaked in their first year in school and then gradually declined over the next few years. The empathy levels climbed in the first three years, dropped dramatically when the students started clinical school and then surged again after the second clinical year. There were no links, however, between the stress and empathy levels.
5	WW Suh, SH Cho, JY Yoo, HS Kim, HR Song, WJ Kim, SM Lee and M Hong [43]	2019	Korea	‘The study aimed to investigate the relationship between empathy and psychosocial factors such as burnout, personality, self-esteem, and resilience.’	271 medical students Cross-sectional study Korean edition of the Jefferson Scale of Empathy student version (JSE-S-K), Maslach Burnout Inventory General Survey (MBIGS), NEO Five-Factor Inventory (NEO-FFI), Rosenberg Self-Esteem Scale (R-SES) and Connor-Davidson Resilience Scale (CD-RISC)	Conscientiousness, depersonalization, personal accomplishment, self-esteem and sex all influenced empathy. Of these, conscientiousness, depersonalisation, personal accomplishment and self-esteem significantly affected empathy.
6	P Rahmatpour, M Chehrzad, A Ghanbari and SR Sadat-Ebrahimi [44]	2019	Iran	‘We aimed to explore the incidence of academic burnout status and its associated factors among [the] Guilan University of Medical Sciences students.’	303 medical science students Cross-sectional study Maslach Burnout Inventory‑Student Survey (MBI‑SS)	Academic burnout in students was found to be substantially associated with marital status, GPA (grade point average), interest in the study field and study time. Students who are disappointed and unhappy due to academic burnout are less likely to participate in class activities, resulting in further academic burnout and lower educational success.
7	H von Harscher, N Desmarais, R Dollinger, S Grossman and S Aldana [45]	2018	USA	‘To understand the relationship between empathy (empathic concern [EC] and personal distress [PD]) and burnout in medical students’	353 medical students Longitudinal relational study Maslach Burnout Inventory (MBI) and Davis Interpersonal Reactivity Scale (IRI)	Over the course of three years, it was observed that the students with high levels of EC had statistically lower burnout scores whereas the students with high levels of PD had statistically higher burnout scores. In this study, EC was found to be associated with lower burnout and PD was found to be associated with higher burnout.
8	S Ebrahimi and F Atazadeh [46]	2018	Iran	‘This study aimed to determine the prevalence of burnout among [the] medical students of Shiraz University of Medical Sciences at the clinical level and its relationship with professionalism.’	230 medical students Cross-sectional study Dimensions of professionalism questionnaires (American Board of Internal Medicine [AIBM] questionnaire), Maslach Job Burnout Inventory	Burnout and professionalism were found to have a negative association with each other.
9	D Richardson, SM Jaber, S Chan, MT Jesse, H Kaur and R Sangha [47]	2016	USA	‘To determine how self-compassion and empathy might influence the degree of burnout, secondary traumatic stress and compassion satisfaction among medical students and residents’	88 medical students and residents Cross-sectional study Professional Quality of Life Scale (burnout, secondary traumatic stress and compassion satisfaction), Neff’s Self-Compassion Scale and the empathic concern and personal distress subscales of the Interpersonal Reactivity Index	Compassion for oneself and for those under one’s care reduces burnout and improves work satisfaction.
10	KH Park, DH Kim, SK Kim, YH Yi, JH Jeong, J Chae, J Hwang and H Roh [48]	2015	Korea	‘To examine the relationship between stress, social support, and empathy among medical students’	2,692 medical students Cross-sectional, relational study Jefferson Scale of Empathy, Perceived Stress Scale and Multidimensional Scale of Perceived Social Support	The stress and social support levels of all the students were found to be strong predictors of empathy. Furthermore, among the female students, there was no significant link between empathy and stress, and among the female students and first-year students, empathy was not significantly predicted by stress.
11	HB Paro, PS Silveira, B Perotta, S Gannam, SC Enns, RR Giaxa, RF Bonito, MA Martins and PZ Tempski [49]	2014	Brazil	“We aimed to assess medical students’ empathy and its associations with gender, stage of medical school, quality of life, and burnout.”	1,350 medical students Cross-sectional, multi-centric, relational study Questionnaires on empathy (Interpersonal Reactivity Index), quality of life (The World Health Organization Quality of Life Assessment) and burnout (Maslach Burnout Inventory)	Personal success was found to have the most significant link with reduced personal discomfort among all the variables that were analysed, and it was also a predicting variable for perspective taking. Empathy and burnout were found to have a substantial inverse relationship with each other.
12	SM Hasan, NI Al-Sharqawi, FA Dashti, M Abdulaziz, A Abdullah, M Shukkur, M Bouhaimed and L Thalib [50]	2013	Kuwait	‘To evaluate the level of empathy among [the] medical students in Kuwait University Medical School and its association with sociodemographic factors, stress levels, and personality’	264 medical students Cross-sectional study Jefferson Scale of Physician Empathy (JSPE-S) medical student English version, Zuckerman-Kuhlman Personality Questionnaire (ZKPQ-50-CC) and Perceived Stress Scale 10-item version (PSS-10)	There was no statistical link between academic achievement and degree of empathy. Students with a higher stress level scored higher on the empathy scale; thus, stress level was found to be significantly and positively connected with empathy.
13	LN Dyrbye, W Harper, C Moutier, SJ Durning, DV Power, FS Massie, A Eacker, MR Thomas, D Satele, JA Sloan, et al [31].	2012	USA	‘The study simultaneously explores the relationship between positive mental health and burnout with professionalism and personal experience.’	2,682 medical students Multi-institutional, exploratory relational study Mental Health Continuum Short Form (MHC-SF), Maslach Burnout Inventory (MBI), Medical Students’ Attitudes toward Providing Care for the Underserved (MSATU) instrument	Positive mental health was found to mitigate some of the negative effects of burnout. Non-flourishing students were more likely to have acted in an unprofessional manner (i.e., cheating and dishonest behaviours). Except for one of the seven behaviours analysed (signing an attendance sheet for a friend who was not present), mental health status did not correlate with professional conduct among the students with burnout. In general, a higher percentage of the flourishing students agreed with the five altruistic professional views about physicians’ societal responsibilities. As the students’ mental health improved, the prevalence of each professional belief grew as well. Three of the five categories involving physicians’ societal responsibilities were found to have a statistically significant relationship with mental health in the students with professional burnout. These findings imply that good mental health may help mitigate some of the detrimental effects of burnout, particularly in terms of certain characteristics of professionalism.
14	CM Brazeau, R Schroeder, S Rovi and L Boyd [51]	2010	USA	‘Medical student burnout is prevalent, and there has been much discussion about burnout and professionalism in medical education and the clinical learning environment. Yet, few studies have attempted to explore [the] relationships between those issues using validated instruments.’	105 medical students Relational study Maslach Burnout Inventory, Jefferson Scale of Physician Empathy – Student Version and the professionalism climate instrument	Burnout, empathy and professionalism climate were all found to be linked with each other. The empathy scores of the people who were burnt out were lower. The burnout scores were found to have substantially significant negative relationships with the professionalism climate scores. Students that are burnt out have a pessimistic outlook and are more inclined to doubt the professionalism of other students and of the residents and instructors in the learning environment; this attitude can also affect what they will learn. The students’ empathy levels were linked with their professionalism.
15	LN Dyrbye, FS Massie, Jr., A Eacker, W Harper, D Power, SJ Durning, MR Thomas, C Moutier, D Satele, J Sloan, et al [52].	2010	USA	‘To determine the relationship between measures of professionalism and burnout among US medical students’	2,682 medical students Multi-institutional, cross-sectional study Maslach Burnout Inventory (MBI), 2-item Primary Care Evaluation of Mental Disorders (PRIMEMD), psychometrically sound Medical Outcomes Study Short Form (SF-8, range 0–100), Medical Students’ Attitudes toward Providing Care for the Underserved (MSATU) instrument, American Medical Association (AMA) Ethical Guidelines of Gifts to Physicians from Industry	Burnout was linked with unprofessional behaviour and a lack of altruistic professional attitude among medical students. It was also found to be linked with self-reported cheating and dishonest clinical behaviour and to be inversely related to altruistic professional values related to physicians’ societal responsibilities.
16	MR Thomas, LN Dyrbye, JL Huntington, KL Lawson, PJ Novotny, JA Sloan and TD Shanafelt [53]	2007	USA	‘To determine whether [the] lower levels of empathy among a sample of medical students in the United States is associated with personal and professional distress and explore whether a high degree of personal wellbeing is associated with higher levels of empathy’	1,098 medical students Multi-institutional, cross-sectional survey Interpersonal Reactivity Index (IRI), Maslach Burnout Inventory and Linear Analogue Self-Assessment (LASA), a 10-item questionnaire measuring quality of life	Empathy was found to be inversely connected to the burnout domains. Medical student empathy was found to be linked with both discomfort and well-being. Rather than advancement through the training curriculum alone, the decline in empathy was found to be linked with student discomfort and quality of life. Burnout in the workplace may be more closely linked with loss of empathy than sadness is.

### Stage 5: Collating, summarising and reporting the results

We recognised thematic groupings in the literature, which included multiple sections such as methods, evidence, defining results and implications. When all the data had been grouped and some initial data had been recognised, we convened an online meeting to discuss the data analysis and interpretation approaches to be used and how we would discuss and reflect these in our manuscript. Our data scrutiny approach mainly involved qualitative thematic analysis. To incorporate a statement of the evidence and the possible inconsistencies in the existing knowledge, interventions and other findings with similar characteristics were thematically organised. Our use of this method facilitated our data collection and made it more structured, helping us identify major themes (mental wellbeing and medical professionalism), significant findings and explicit information pertinent to our research question (to be explained in the subsequent sections).

### Reflections on the process of critical evaluation

In my capacity as the initial author and as one of the primary reviewers (KS), I felt compelled to share some of my thoughts regarding the procedure of carrying out the critical evaluation.

MSBY and I discussed multiple times over 6 months. First, research articles were chosen. This crucial step involved phases of independently evaluating abstracts, comparing selections, and debating disagreements. The second stage was similar in that we independently read 16 full-text articles, took notes on them, and then start to produce a synthesized evaluation.

As a doctorate student doing qualitative research, I had less experience than the second author and my main supervisor MSBY; this altered our early work dynamics. Knowing this and exercised thoughtfulness while expressing my own voice, but the second reviewer generously allowed me to disagree when essential. Early in the analysis, we interpreted the framework together. I gained self-confidence, and I became more comfortable voicing my views and judgments. As our task tends to be critical, having a senior colleague as a second reviewer brings an expert voice into the argument and decision-making process. This supplemented a wonderful strength, especially during the collaborative synthesis of the included documents.

WNA/MAMY/MZMN also contributed with their experience. In the beginning, we relied on their clinical and academic experience to resolve differences. This happened at a few stages of the research process, such as when formulating search phrases to retrieve as many prospective documents as feasible. Moreover, at the end of my research, I had a dilemma about how to present the results, which I overcame with the help of the second reviewer and my academic main supervisor (MSBY). I believe that research can be full of such challenges yet reflexivity goes a long way toward successfully solving the inevitable dilemma in the course of research tasks. We encourage others to take reflexive steps in a pattern similar to our own.

## Results

### Descriptive statistics and study characteristics

All the initially obtained articles (13,076) were reviewed partly (title and abstract) or in full, and 16 articles were finally deemed eligible to be included in our scoping review (Figure 1). The studies reported in such articles were conducted across nine countries, with a large proportion conducted in the United States (*n* = 6) [31,45,47,52–54], two in Iran [44,46], two in Korea [43,48] and one each in Indonesia [42], China [41], India [40], Germany [39], Brazil [49] and Kuwait [50]. These nine countries are situated in four continents: North America, Asia, Europe and South America. Most of the studies (*n* = 12) utilised a cross-sectional survey [40–50,52,53], with two using a longitudinal survey [39,45] and two, a relational survey [31,54].

Among the many inventories/survey tools that were used by the studies included in our review, the most used (used by more than one study) are listed in the following table.

### Results of the thematic analysis

The studies included in our review helped us find a fitting answer to our research question by using extracted points relating to the two main study themes: mental wellbeing and medical professionalism. These two themes embody the most discussed issues about the relationships between mental wellbeing constructs and various medical professionalism attributes in the undergraduate medical education setting within the relevant literature.

**Figure 2.**
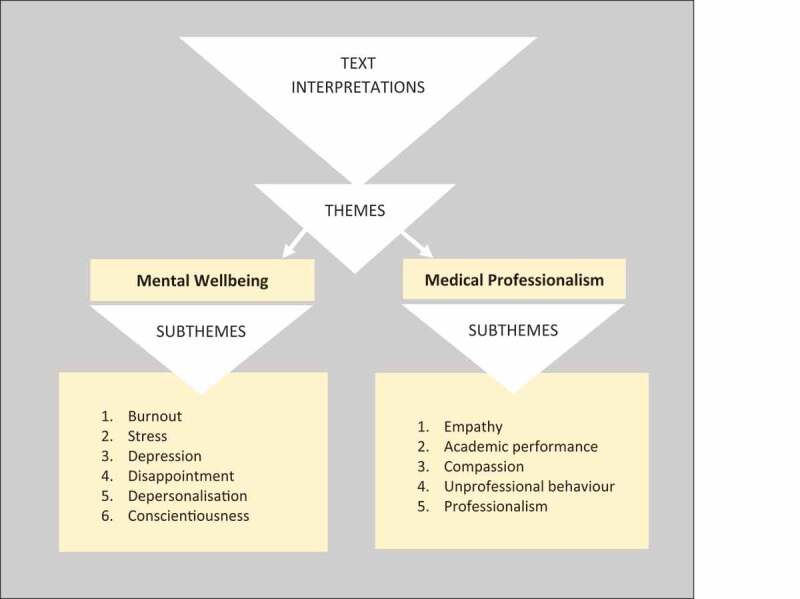
Graphic representation of themes and subthemes yielded as a result of text interpretations.

The recognised themes brought forth subthemes (see Figure 2). Under theme 1 (mental wellbeing), six subthemes emerged within the studies included in our review: burnout [31,40,41,44–47,49,52–54], stress [42,48], depression [39], disappointment [44], depersonalisation and conscientiousness [43]. Theme 2 (medical professionalism), on the other hand, had five subthemes: empathy [39,41–50,53,54], academic performance [40,50], compassion [47], unprofessional behaviour [31,52] and professionalism [46].

### Association between burnout and empathy

**Figure 3.**
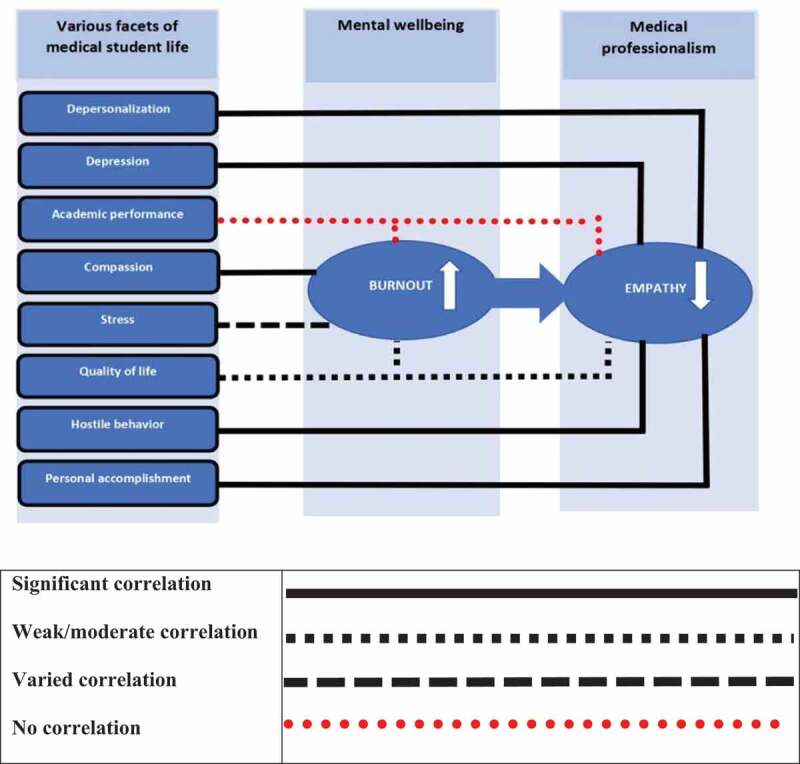
Illustrative depiction of the interrelationship model of mental well-being and medical professionalism attributes.

Most of the studies included in our review (*n* = 11; 68.75%) addressed burnout, and Maslach Burnout Inventory (MBI) was used by 10 of such studies (Table 3) whereas one study utilised Copenhagen Burnout Inventory (CBI). Increased level of burnout resluted a decline in the empathy (Figure 3). On the other hand, empathy was the most targeted subtheme of MP as most of the studies included in our review (*n* = 11) targeted it, and the most used inventory was Jefferson Scale of Empathy (JSE).

**Table 3.** The most utilised data collection tools within the included studies.

Inventory/survey	Studies
Maslach Burnout Inventory	[31,41,43–46,49,52–54]
Jefferson Scale of Empathy	[39,42,43,48,50,54]
Interpersonal Reactivity Index	[41,47,49,53]
Perceived Stress Scale	[42,48,50]
Medical Students’ Attitudes toward Providing Care for the Underserved (MSATU) instrument	[31,52]

Empathic concern is associated with warm, compassionate feelings toward people in distress, whereas personal distress is associated with feeling others’ sadness and discomfort through observation of their negative experiences [55]. Table 1 shows that the results of an included study [45] indicating that empathic concern was correlated with lower burnout scores. In contrast, the other type of empathy, personal distress empathy, is linked with higher burnout scores. Furthermore, there was no significant difference across the empathic concern dimension’s three years of medical training. At the same time, the median scores for the personal distress dimension indicated no significant difference across years. Empathy and burnout have a significant inverse association with each other, as reported by Paro *et al* [49], and overall, the empathy scores were weakly correlated with quality of life and only moderately correlated with burnout. We also found out that professional burnout could be more closely related to deteriorating empathy than depression is. Furthermore, burnout was inversely correlated with empathy (depersonalisation with empathy independent of gender). In contrast, the students’ sense of personal accomplishment was found to be positively correlated with empathy independent of gender (all *p* < 0.001) [53].

### Association between stress and empathy

Table 1 also shows that the studies included in our review reported no correlation [42] between stress level and empathy level; however, stress and social support were reported to be significant forecasters of empathy among all the students [48]. SM Hasan, NI Al-Sharqawi, FA Dashti, M Abdulaziz, A Abdullah, M Shukkur, M Bouhaimed and L Thalib [50], on the other hand, reported that stress level is meaningfully linked with empathy as the students in their study who had advanced stress levels recorded higher scores on the empathy scale. Besides, a substantial association was found between aggressiveness and empathy as the students with hostile behaviours scored lower on the empathy scale than the students with non-hostile behaviours did.

## Discussion

We’re discussing mental health and medical professionalism. Professionals must be ethical and deliver great work. It implies a calm expert in control of the situation and his or her emotions. Professionalism seeks excellence and sets high standards and goals. Reasonable people shouldn’t expect less. Professional perfectionism can lead to fatigue, stress, despair, and decreased academic achievement and productivity. This means that professionals suffering from compromised mental wellbeing can no longer devote themselves psychologically to their work, are pessimistic and cynical about their clients (patients) and have an unfavourable view of their work. Frustration, anger, exhaustion and a lack of enjoyment of or reward from one’s work are all examples of possible burnout symptoms [56]. Poor mental wellness, characterized by fatigue, stress, depression or anxiety, and poor quality of life, was connected with lesser medical professionalism, such as empathy.

Empathy is described as a central characteristic of altruistic healthcare providers [57] and is also one of the three fundamental professional principles outlined in the Physician Charter (2002) [58]. It is a critical element of medical professionalism. Various groups, such as doctors, patients and students, consider empathy a core value of a good doctor [59]; hence, if it declines, it could also cause a decline in medical professionalism.

The studies included in our review [41,44,45,49,53,54] provided evidence of a relationship between burnout features and empathy. A link between depression scores and JSES scores was reported, and lower scores for depression were also related to advanced JSE-S sum scores. These results are aligned with those reported by MR Thomas, LN Dyrbye, JL Huntington, KL Lawson, PJ Novotny, JA Sloan and TD Shanafelt [53] and confirm the results on the adverse association between depression and empathy among physicians and medical students. Among medical students and health professionals, burnout has a considerable negative correlation with life satisfaction. We also found negative associations between burnout and empathy [45,49,51,53], altruism [52] and professionalism [46,51], and these were consistent with the findings of numerous studies [60–67]. However, it was reported that empathy does not have any correlation with stress level [42] and that medical students’ stress levels are most significant in their first year of study and show a tendency to decline over the subsequent years. The students’ empathy levels were amplified during their first three years, declined meaningfully when they entered the first clinical year and were amplified again during their second clinical year. A drop in empathy scores in the later clinical years was also reported in another study [68]. On the other hand, interestingly, stress level was meaningfully linked with empathy as the students with higher stress levels scored higher on the empathy scale [50].

Regarding academic performance, we found that awareness of one’s academic performance and disappointment with one’s performance in the professional examination are not linked with burnout [40]. Likewise, there is no numerical relation between academic performance and empathy level [50]. This contrasted with the previous reports [69].

Conscientious physicians have been demonstrated to have superior clinical ability [70], and clinical competence and empathy have been shown to be positively connected to each other [71]. Additionally, because conscientious individuals are more likely to actively seek to resolve problems with others and less likely to have conflicts with others compared to non-conscientious individuals, they may be viewed as having more empathy than the latter [72]. With regard to the factors that influence medical students’ empathy, it was discovered that conscientiousness, depersonalisation, personal accomplishment and self-esteem substantially affect such students’ empathy [43].

Unprofessional conduct in medical school is a good predictor of unprofessional conduct in practice Our examination of the papers included in our review found that burnout is linked to unprofessional conduct [31]. Similar findings were reported, were burnout was linked with their self-reported unprofessional demeanour [73] insensitive patient care practices, declined empathy and altruism, cheating and fraudulent behaviors [74,75]. Therefore, burnout may be an added significant variable causing unprofessional behaviour. Additionally, it was suggested that positive mental health could reduce some adverse outcomes of burnout, particularly those related to specific characteristics of professionalism.

## Future directions

This scoping review also revealed the relevant research gap in terms of explicit primary assessment of cause-effect relationship between mental wellbeing and medical professionalism and suggests a need for future studies. A systematic review with a methodological quality appraisal is recommended. Explicit primary research may also be appropriate for assessing and improving the state of medical professionalism in the context of its association with mental health issues, and may suggest what could address such academic challenge.

## Strengths and limitations

To be transparent and reproducible, this study used a well-known and commonly accepted approach for scoping several articles to synthesise the data available from them and to clarify the critical link between medical professionalism and mental wellbeing through an exhaustive data search. We took steps to minimise error and boost dependability (e.g., by involving several reviewers) and to ensure that data are retrieved, extracted and presented systematically. Nevertheless, our study had limitations, and we cite them here. The quality of the evidence was not formally evaluated, and data were gathered from studies with various research designs and approaches, resulting in a relatively large number of studies. We believe medical professionalism’s core traits remain similar yet being context specific, expressions for its core values might differ in different geographic locations. Hence, instead of an overview of the available relevant literature, we focused on and summarised the relevant studies pertaining to our research question. Finally, as we could work only with studies written in English, we omitted foreign-language studies, which may have influenced our inferences.

## Conclusion

In this scoping review, we gathered data that could shed light on the relationship between medical professionalism and mental wellbeing in the undergraduate medical education setting, an area with developing evidence for both the association between these two domains and the risks linked with such association. Compassion for oneself and for others in one’s care was found to relieve burnout and increase professional satisfaction. Additionally, the medical professionalism attributes were found to decline with growing mental wellbeing issues, and a significant inverse association was found between empathy and burnout. This has a great potential to harm medical students’ overall health and learning capabilities at present and their mental attitude towards their patients in the future. Indeed, medical students’ learning could affect the way they would attend to their patients in the future, which could affect the future society. Therefore, identifying such association and its related effects is a critical area of concern in the medical education setting, and an enhanced understanding of the problem is required. Although, it can be difficult to develop a comprehensive program for teaching professionalism at all levels at the same time, yet one should begin with those basic activities that are already dedicated to teaching professionalism (e.g., cognitive base targeting a precise outline of definitions and attributes of the professional). Similarly at risk students (exhibiting decline in professionalism attributes (e.g., empathy) and, or mental wellbeing issues (ee.g.,burnout) must be provide with opportunity for academic and personal counselling.

## Availability of data and materials

All the data that were generated or analysed during the study were included in this article and in its supplementary information files.

## Appendix

**Table:**

Appendix A		
Details of finalised search strategy and tools utilised 1986–2021		
DATABASE	SEARCH TERMS/STRINGS	DOCUMENTS RETRIEVED
Web of Science	ALL=(‘mental wellbeing’ OR ‘medical professionalism’ AND ‘undergraduate’ AND ‘student’) Refined by: Document type: Articles Language: English Publication year: 1986–2021 Web of Science category: Psychology Clinical or Psychiatry QUERY LINK: https://www.webofscience.com/wos/woscc/summary/dea1a98d-7a37-4a10-822c-cca81ef811ac-10699566/relevance/1	n = 6,234
PubMed	(((((((((((”Health”[Mesh]) OR “Health/psychology”[Mesh]) OR (”Mental Health/standards”[Mesh] OR “Mental Health/trends”[Mesh])) OR “Burnout, Professional”[Mesh]) OR “Occupational Stress/education”[Mesh]) AND (”Professionalism/education”[Mesh] OR “Professionalism/standards”[Mesh] OR “Professionalism/trends”[Mesh])) OR “Ethics, Medical”[Mesh]) OR “Professional Misconduct/trends”[Mesh]) OR “Professional Competence”[Mesh]) AND “Students, Medical”[Mesh]) AND “Education, Medical, Undergraduate”[Mesh]) OR (”Education, Medical, Undergraduate/standards”[Mesh] OR “Education, Medical, Undergraduate/trends”[Mesh]) **Filters**: Journal article, within the period from 1986 to 2021	n = 5,795
ScienceDirect	‘mental AND wellbeing’ AND ‘medical AND professionalism’ AND ‘undergraduate’ AND ‘medical AND student’ Published within the period from 1986 to 2021	n = 629 This search history was saved on the ScienceDirect website (My search history).
Scopus	ALL (‘mental AND wellbeing’ AND ‘medical AND professionalism’ AND ‘undergraduate’ AND ‘medical AND student’)	n = 418
